# Dual promotional effect of Cu_*x*_O clusters grown with atomic layer deposition on TiO_2_ for photocatalytic hydrogen production†

**DOI:** 10.1039/d2cy00400c

**Published:** 2022-06-08

**Authors:** Saeed Saedy, Nico Hiemstra, Dominik Benz, Hao Van Bui, Michael Nolan, J. Ruud van Ommen

**Affiliations:** Department of Chemical Engineering, Delft University of Technology Van der Maasweg 9 2629 HZ Delft The Netherlands s.saedy@tudelft.nl; Faculty of Materials Science and Engineering, Phenikaa University Yen Nghia, Ha-Dong District Hanoi 12116 Vietnam; Tyndall National Institute, University College Cork Lee Maltings, Dyke Parade T12 R5CP Cork Ireland

## Abstract

The promotional effects on photocatalytic hydrogen production of Cu_*x*_O clusters deposited using atomic layer deposition (ALD) on P25 TiO_2_ are presented. The structural and surface chemistry study of Cu_*x*_O/TiO_2_ samples, along with first principles density functional theory simulations, reveal the strong interaction of ALD deposited Cu_*x*_O with TiO_2_, leading to the stabilization of Cu_*x*_O clusters on the surface; it also demonstrated substantial reduction of Ti^4+^ to Ti^3+^ on the surface of Cu_*x*_O/TiO_2_ samples after Cu_*x*_O ALD. The Cu_*x*_O/TiO_2_ photocatalysts showed remarkable improvement in hydrogen productivity, with 11 times greater hydrogen production for the optimum sample compared to unmodified P25. With the combination of the hydrogen production data and characterization of Cu_*x*_O/TiO_2_ photocatalysts, we inferred that ALD deposited Cu_*x*_O clusters have a dual promotional effect: increased charge carrier separation and improved light absorption, consistent with known copper promoted TiO_2_ photocatalysts and generation of a substantial amount of surface Ti^3+^ which results in self-doping of TiO_2_ and improves its photo-activity for hydrogen production. The obtained data were also employed to modify the previously proposed expanding photocatalytic area and overlap model to describe the effect of cocatalyst size and weight loading on photocatalyst activity. Comparing the trend of surface Ti^3+^ content increase and the photocatalytically promoted area, calculated with our model, suggests that the depletion zone formed around the heterojunction of Cu_*x*_O–TiO_2_ is the main active area for hydrogen production, and the hydrogen productivity of the photocatalyst depends on the surface coverage by this active area. However, the overlap of these areas suppresses the activity of the photocatalyst.

## Abstract 2

The depletion zone formed around the Cu_*x*_O clusters is the main photocatalytically active area, and the H_2_ production rate depends on surface coverage with this area; however, the overlap of these areas suppresses the photocatalyst activity.

## Graphical abstract

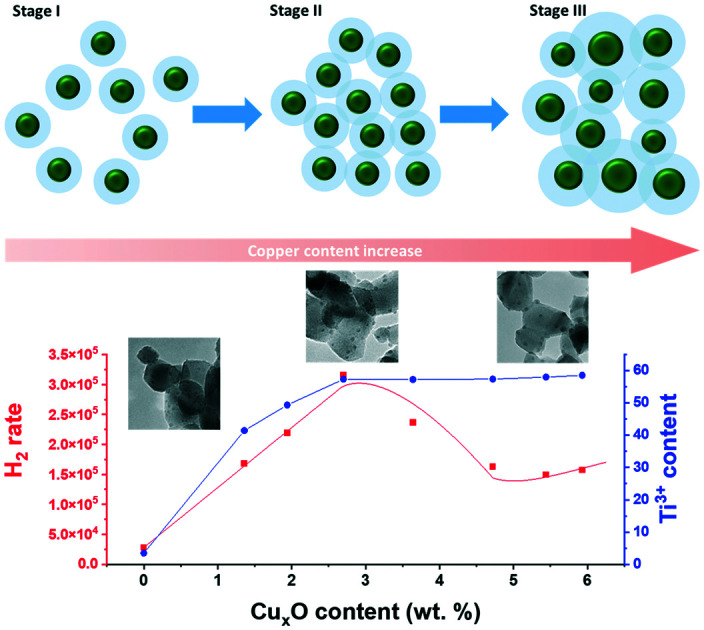

## Introduction

Climate change and its devastating environmental effects constitute the major long-term threats that our planet is facing. On the other hand, the depletion of fossil fuel reserves is a big challenge to the energy security of our society. It is anticipated that the annual energy consumption will be doubled by 2050 compared to 2015.^1^ Relying on fossil fuels may partly solve this issue from an energy supply perspective; however, it will further escalate the environmental crisis *via* enormous CO_2_ release and that of other harmful emissions such as NO_*x*_ and SO_*x*_.^2^ The use of hydrogen as a carbon-free energy carrier is a promising solution, offering a sustainable energy system.^3^ Hydrogen is potentially the most sustainable and cleanest transportation fuel^4,5^ and is already used in fuel cells. It produces no pollutants nor greenhouse gases while having a high energy capacity.^5^ The primary advantage of hydrogen is that it can be transported and stored with minor losses using the current chemical fuel infrastructure with some modifications,^6^ making a smooth transition from fossil fuels possible. Nowadays, the majority of hydrogen, *c.a.* 95%, is produced from fossil fuels *via* processes like methane reforming, coal gasification, *etc.*^7^ and is known as grey hydrogen. These processes are not environmentally friendly, use fossil fuels and contribute to significant CO_2_ emissions, so that the hydrogen obtained in this way is unsustainable.

The solar energy arriving at the earth's surface every hour is enough for one year of global consumption.^2^ The photocatalytic (solar) production of hydrogen is regarded as a cost-efficient and sustainable approach for hydrogen production that enables harvesting of sunlight and long-term storage of the most abundant and renewable energy source available.^2,3,8^ Since the first publication from Fujishima and Honda^9^ on photolytic water splitting using titanium dioxide (TiO_2_), there has been significant research on solar hydrogen production, and it is well-reviewed.^2,3,5–7,10^ Among the different photocatalytically active materials, TiO_2_ shows superiority for solar hydrogen production due to its unique properties, including high chemical stability, non-toxic nature, environmental compatibility, availability, and low cost.^3,5,10^ However, TiO_2_ suffers from a wide bandgap, which is larger than 3 eV for its different crystal structures, making absorption and utilization of UV light dominant rather than absorption of visible light.^2,11^ It also suffers from significant electron–hole recombination, reducing its photocatalytic efficiency.^3^

There are several approaches to improve the photocatalytic efficiency of TiO_2_, which mainly focus on bandgap narrowing to activate it for visible light and enhance the electro-hole separation.^3,5^ Doping non-metal dopants like nitrogen, sulfur, and phosphorous, and surface decoration/doping using metal/metal oxide nanoparticles (NPs) such as copper, iron, silver, gold, and platinum are some of the more widely studied attempts to improve the photocatalytic activity of TiO_2_.^2,3,5^ Additionally, self-doping of TiO_2_ by Ti^3+^ also improves its photo-activity for hydrogen production.^12–14^ Among these photocatalytic activity promoters, copper is a promising candidate for promoting solar hydrogen production due to its low cost, narrow bandgap, and comparable cocatalyst activity to expensive promoters such as gold and silver.^3^ Copper oxide is a narrow bandgap p-type semiconductor that, in combination with the n-type TiO_2_, improves its visible light absorption and produces a p–n junction, enhancing the overall photocatalytic activity toward hydrogen production.^3,15–22^

The promotional effects in the cocatalyst-promoted TiO_2_ systems are well studied, and numerous attempts have been made to describe the effect of cocatalyst loading on the photocatalytic activity of TiO_2_, suggesting the existence of an optimum point for cocatalyst loading.^23–28^ A fundamental understanding of the correlation between the cocatalyst particle size/loading and the photocatalytic activity of the photocatalyst will pave the way for the design, development, and large-scale production of a high-performance photocatalyst. Recently, Mills *et al.*^29^ developed a model which they term “expanding photocatalytic area and overlap (EPAO)” based on the previously developed metal support interface (MSI) model by Bowker *et al.*^30^ to describe the relationship between the hydrogen production rate and the cocatalyst loading during photocatalytic methanol reforming. The EPAO model is built upon six assumptions^29^ and is validated for Pd/TiO_2_ and Pt/TiO_2_ systems, suggesting that hydrogen production rate is proportional to the total photocatalytically active area surrounding the cocatalyst particle; it also suggests that the overlap of these active areas at high coverage results in a drop in hydrogen production. The EPAO model makes the quantification of the relation between the cocatalyst loading and the photocatalyst activity possible. In this paper, we seek to further improve the EPAO model using copper oxide modified TiO_2_ where copper oxide is deposited by atomic layer deposition (ALD).

Developing an insightful understanding that can lead us to a precise model to describe the correlation between the cocatalyst particle size/loading and the photocatalytic activity requires a set of catalysts with a well-defined structure. Such catalysts are usually developed for surface science studies on single crystal flat substrates; however, translating those systems into real catalytic systems is quite challenging. On the other hand, a catalyst prepared using the conventional liquid phase methods such as impregnation and precipitation lacks such a well-defined structure.^31,32^ Atomic layer deposition is a leading method for synthesizing well-defined advanced functional nanomaterials and can be employed to surmount this issue. As an excellent technique for supported NPs synthesis, ALD makes the precise deposition of well-controlled particles in terms of size and composition possible; it also allows controlling the amount of material deposited on the substrate at the atomic level.^31,33–35^ The possibility of depositing uniform NPs on the support with controlled size, shape, and morphology using ALD provides an excellent opportunity to obtain high-activity photocatalysts with a well-controlled structure. The advantages of ALD as a novel method for preparing supported catalysts are well-reviewed and addressed in multiple works.^31,36–38^

In this work, we aim to elucidate the correlation between the cocatalyst particle size/loading of Cu_*x*_O on TiO_2_ and the photocatalytic activity during solar hydrogen production. We employed ALD to deposit Cu_*x*_O NPs on AEROXIDE® P25 TiO_2_ NPs with precise control over loading and Cu_*x*_O particle size. The obtained samples were used as photocatalysts for the solar production of hydrogen from photolysis of a methanol/water solution, showing superior hydrogen productivity compared to pristine TiO_2_. The XPS analysis revealed Ti^4+^ reduction to Ti^3+^ after copper ALD, with an initial linear trend with copper loading, and plateauing of Ti^3+^ content at about 2.3 wt% of copper. This is the copper content at which hydrogen productivity is maximum. The modified EPAO model was validated using these results with a good fit. The model indicates the development of a photocatalytically promoted area (PPA) around Cu_*x*_O clusters, revealing a dual promotional effect of ALD grown Cu_*x*_O clusters: increased charge carrier separation and improved light absorption, known for copper promoted TiO_2_ photocatalysts, and generation of a substantial amount of surface Ti^3+^, leading to self-doping of TiO_2_. Complementary first-principles density functional theory (DFT) simulations are also utilized to understand the interaction of Cu_*x*_O clusters with the TiO_2_ support and assess the origin of titanium and copper oxidation states. The DFT results align with our experimental observations, indicating the stabilization of Cu_*x*_O clusters on TiO_2_, and a partial reduction of Ti^4+^ to Ti^3+^ due to the interaction of Cu_*x*_O clusters with TiO_2_.

## Experimental

### Materials

Evonik P25 powder (Evonik Industries – Hanau, Germany), containing TiO_2_ NPs with an average diameter of ∼21 nm and a specific surface area of ∼50 m^2^ g^−1^, was employed as substrate for copper deposition. While P25 is a complex mix of rutile and anatase, it is a standard photocatalytically active TiO_2_ material that has been widely used in many studies of photocatalysis, offering also low cost, large scale availability, and higher activity than either rutile or anatase individually. Copper(i) hexafluoropentanedionate-vinyltrimethylsilane (Cu(i)(hfac)(TMVS)) complex was purchased from Advanced Tech. & Ind. Co., Ltd, and used as the CuO_*x*_ precursor.

### Atomic layer deposition of copper(i/ii) oxide

The CuO_*x*_/TiO_2_ samples were synthesized following the procedure described in our previous work.^39^ In summary, a vibration-assisted fluidized bed reactor^34,40^ operating at atmospheric pressure was employed to deposit Cu_*x*_O ultrafine clusters on P25 NPs. Cu(i)(hfac)(TMVS) evaporated at 60 °C was delivered to the ALD reactor, heated up to 250 °C using an IR lamp, while water evaporated at room temperature was used as the second reactant. Nitrogen (99.999 vol%) was used as the carrier gas to deliver ALD reactants and purge the reactor. Typically, 1.5 g of TiO_2_ powder was fluidized using 0.5 L min^−1^ nitrogen stream (superficial gas velocity of ∼1.7 cm s^−1^); and a combination of different pulse times of Cu(i)(hfac)(TMVS) and water, and different ALD cycle numbers were employed to obtain Cu_*x*_O particles on TiO_2_ with a well-controlled control particle size. Table 1 summarizes the details of the synthesis condition used to prepare the different samples in this study.

**Table 1:** The ALD synthesis parameters, used for Cu_*x*_O ALD on P25 TiO_2_ nanopowder

Sample	Cu(i)(hfac)(TMVS) pulse (min)	Purge 1 (min)	Water pulse (min)	Purge 2 (min)	Number of cycles
1	2.5	5	10	5	10
2	5	5	5	5	10
3	5	5	10	5	10
4	10	5	2.5	5	10
5	10	5	10	5	10
6	5	5	5	5	20
7	30	5	10	5	10

### Characterization

The copper loading on ALD synthesized CuO_*x*_/TiO_2_ samples was analyzed using inductively coupled plasma-optical emission spectrometry (ICP-OES) method employing a PerkinElmer Optima 5300 DV ICP-OES instrument. Typically, about 25 mg of sample was digested in 4.5 ml 30% HCl + 1.5 ml 65% HNO_3_ + 0.2 ml 40% HF while microwave irradiation. The digestion time in the microwave was 60 min with the radiation power of 1300 W. The digested samples were diluted to 50 ml with Milli-Q water prior to measurement.

The transmission electron microscopy (TEM) images of the ALD deposited Cu_*x*_O clusters on P25 TiO_2_ were acquired using a JEOL JEM1400 microscope operating at a voltage of 120 kV working in bright-field mode. The Cu_*x*_O/TiO_2_ particles were dispersed in ethanol *via* sonication in an ultra-sound bath and transferred onto Quantifoil copper TEM grids (coated with perforated carbon). The particle size of individual particles was measured using ImageJ software. The average particle size and particle size distribution (PSD) curves were obtained using the size of more than 350 individual particles measured using ImageJ.

The surface chemistry of Cu_*x*_O/TiO_2_ samples was studied using a Thermo Scientific™ K-Alpha™ X-ray photoelectron spectrometer. The monochromated X-ray with a spot of 400 μm was generated using aluminum Kα radiation (photon energy of 1486.7 eV), and the differential charging was compensated using a flood gun. A step size of 0.1 eV was used for acquiring the high-resolution scans. The CasaXPS software was employed to analyze the obtained XPS spectra, and the positions of peaks were calibrated using the aliphatic carbon 1s peak (284.8 eV).

The crystallinity of the samples was investigated using a Bruker D8 Advanced diffractometer with Bragg–Brentano geometry, equipped with a Cu-Kα radiation source (Cu radiation wavelength: Kα_1_(100) = 1.54060 Å, Kα_2_(50) = 1.54439 Å) working at 40 kV and 25 mA and a Lynxeye-XE-T position-sensitive detector. The X-ray diffraction (XRD) patterns were acquired in the 2*θ* range of 10–90° with a fixed sample illumination area (18 × 5 mm^2^), and a step size of 0.008° and a measuring time of 0.15 s per step were employed.

### Photocatalytic hydrogen evolution reaction

The photocatalytic hydrogen evolution reaction (HER) activity of Cu_*x*_O/TiO_2_ samples was evaluated using a photocatalytic setup described in detail elsewhere.^8^ The Cu_*x*_O/TiO_2_ photocatalyst samples were suspended (0.2 g L^−1^) in a 10% v/v methanol in water solution. A custom-made Pyrex reactor with quartz window with a total volume of 42.1 mL, including 17.1 mL headspace and equipped with a water jacket, was employed for photocatalytic HER evaluation under UV-visible light generated using a 500 W Xe/Hg lamp (66983, Newport) without an optical filter. The produced hydrogen was quantified using a CP 9001 gas chromatograph (GC, Chrompack). Typically, the suspension of photocatalyst in methanol–water solution was placed in the photoreactor, and the headspace of the photoreactor was purged with 10 mL min^−1^ argon flow for 30 min while there was no light radiation (under dark condition); after deoxygenating the headspace of the photoreactor, the argon flow was reduced to 1 mL min^−1^ to carry the headspace gas to GC sampling loop. The photoreactor was kept under dark condition for 1 hour while the headspace gas was analyzed using GC, then the photoreactor was illuminated for 20 hours while the HER products were analyzed using GC with 30 min injection intervals. The temperature of the photoreactor was kept at 27 °C, and the photocatalyst was kept suspended in the solution *via* magnetic stirring.

### DFT simulations

Periodic plane wave density functional theory calculations were performed using the (101) surface of anatase TiO_2_ modified with Cu and CuO as a model for the Cu/CuO_*x*_-modified anatase system using the VASP5.3 code.^41,42^ A kinetic energy cut-off of 400 eV is used. The core–valence interaction is described with projector augmented wave (PAW) potentials,^43,44^ with 4 valence electrons for Ti, 6 for O, and 17 for Cu. The Perdew–Burke–Ernzerhof approximation to the exchange–correlation functional was used.^45^ The anatase (101) substrate was modeled as a 4 O–Ti–O trilayer slab, with a (4 × 4) surface supercell expansion (*a* = 21.776 Å, *b* = 15.104 Å) and a vacuum gap of 16 Å. This is the lowest energy surface of anatase TiO_2_. Due to the size of the surface supercell expansion, Γ-point sampling was used, and the convergence criteria for the energy and forces were 10^−4^ eV and 0.02 eV Å^−1^. All calculations were spin-polarized, and there were no symmetry constraints applied. Hubbard U corrections were implemented, with U(Ti) = 4.5 eV, to consistently describe the partially filled 3d state in reduced Ti^3+^.^46,47^ Cation oxidation states were determined from Bader charge analysis and spin magnetizations.

To model the different surface modifications, the following models are used: Cu_20_ cluster on anatase (101), Cu_10_O_10_ on anatase (101), 2 Cu_10_ nanoclusters on anatase (101), and reduced CuO nanoclusters (by removing oxygen from CuO).

The energy gain, *E*^ads^, when the Cu or CuO nanocluster is adsorbed on the anatase (101) surface, is computed from:

1 *E*^ads^ = *E*(CuO_*x*_@TiO_2_) − [*E*(CuO_*x*_) + *E*(TiO_2_)]

where *E*(CuO_*x*_) is the energy of the free copper (*x* = 0) or CuO nanocluster, *E*(TiO_2_) is the energy of the bare anatase (101) slab and *E*(CuO_*x*_–TiO_2_) is the energy of the Cu/CuO_*x*_ nanocluster interfaced with the anatase (101) slab.

The formation energy for oxygen vacancies in CuO_*x*_ is:

2 *E*^ads^[*E*(CuO_*x*−*δ*_@TiO_2_) + 1/2*E*(O_2_)] − *E*(CuO_*x*_–TiO_2_)

where *E*(CuO_*x*−*δ*_@TiO_2_) is the energy of the CuO nanocluster with an oxygen vacancy interfaced with anatase (101), *E*(CuO_*x*_–TiO_2_) is the energy of the CuO_*x*_ nanocluster interfaced with the anatase (101) slab, and *E*(O_2_) is the energy of the free O_2_ molecule. The use of O_2_ as a reference is common in oxide vacancy formation energy calculations and the *δ* in eqn (2) signifies CuO_*x*_ with removed oxygen.

## Results and discussions

We employed ALD synthesis to attain good control over the cocatalyst particle size and dispersion in a wide range of copper loading and obtain a set of well-defined photocatalysts to investigate the correlation between the cocatalyst particle size/loading and the photocatalytic activity. The ALD synthesized Cu_*x*_O/TiO_2_ photocatalysts were characterized using ICP-OES, TEM, and XPS methods and were employed for solar hydrogen production from a methanol/water solution as photocatalysts. DFT simulations also were carried out to develop a better understanding of the interaction of Cu_*x*_O clusters with TiO_2_. The obtained results demonstrated the dual promotional effect of ALD deposited Cu_*x*_O clusters and helped us modify the EPAO model and quantitatively approximate the surface coverage of photocatalytically promoted area by Cu_*x*_O clusters and correlate it to Ti^3+^ content on the surface of Cu_*x*_o/TiO_2_ samples.

### Physiochemical properties of CuO_*x*_/TiO_2_ photocatalysts

**Fig. 1:**
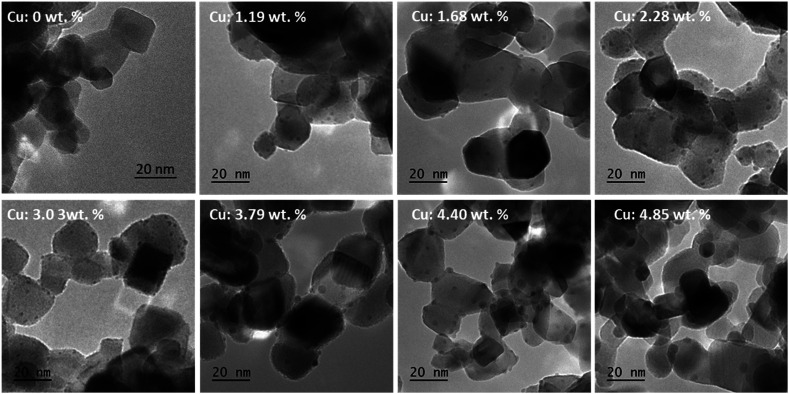
The TEM images of pristine P25 TiO_2_ nanopowder the ALD synthesized Cu_*x*_O/TiO_2_ samples with different copper content.

**Fig. 2:**
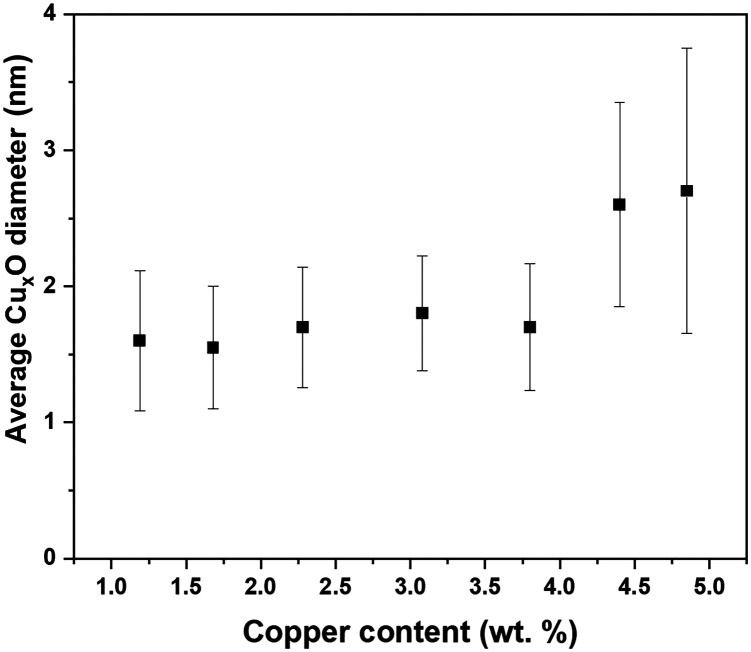
The average Cu_*x*_O particle size as a function of copper loading.

The ALD deposition of p-type Cu_2_O film using Cu(i)(hfac)(TMVS) and water at atmospheric pressure is previously reported by Muñoz-Rojas *et al.*;^48^ additionally we recently reported the self-limiting behavior and ALD deposition of ultra-fine Cu_2_O clusters on P25 TiO_2_ NPs using a fluidized bed reactor operating at atmospheric pressure.^39^ Using the same procedure and applying different precursor/co-reactant pulse times (Table 1), we aimed to control the size of Cu_*x*_O clusters. The ICP-OES analysis indicated copper weight loading in the range of about 1–5%; the exact values are given in Table 2. The TEM images revealed highly dispersed ultra-fine Cu_*x*_O clusters deposited on P25 nanopowder with well-controlled size (Fig. 1). The PSD analysis demonstrates a narrow size distribution for the samples with copper content below 3.79 wt% (Fig. S3†); while the PSD of the samples with 4.40 and 4.85 wt% copper becomes wider and right-skewed. Interestingly, the samples with the copper content of 1.19, 1.68, 2.28, 3.08, and 3.79 wt% have almost the same average Cu_*x*_O size, *i.e.*, ∼1.7 nm with a maximum standard deviation of ±0.5 nm (Table 2 and Fig. 2). The almost constant Cu_*x*_O particle size trend, shown in Fig. 2, for the samples with copper content below 3.79 wt%, which obviously can be seen in TEM images (Fig. 1 and S3†), suggests that the Cu_*x*_O nucleation is dominant until this loading. Therefore, in copper content below 3.79 wt%, further copper added to the surface results in new grown Cu_*x*_O clusters. At copper content higher than 3.79 wt%, the increase of the number of small Cu_*x*_O clusters, and consequently the decrease of distance between them, changes the dominant mechanism from nucleation to particle diffusion and coalescence, resulting in particle size growth and the decrease of the number of particles, since some small particles merge and form larger ones.^49,50^ The decrease of the population of Cu_*x*_O clusters and their growth can be seen in Fig. 1 for samples with the copper content of 4.40 and 4.85 wt%, and the change of PSD graph shape for these two samples is evident in Fig. S3.† The right-skewed PSD graph of these samples (Fig. S3†) also indicates that the dominant growth mechanism for these samples is particle diffusion and coalescence.^49,50^

**Table 2:** The copper content of the ALD synthesized Cu_*x*_O/TiO_2_ samples, obtained by ICP-OES analysis and the average copper particle size obtained by TEM imaging

Sample	Copper loading (wt%)	Average copper particle size (nm)
1	1.19	1.6 ± 0.5
2	1.68	1.5 ± 0.4
3	2.28	1.7 ± 0.4
4	3.08	1.8 ± 0.4
5	3.79	1.7 ± 0.5
6	4.40	2.6 ± 0.7
7	4.85	2.7 ± 1.0

The XRD analysis of the Cu_*x*_O/TiO_2_ samples did not reveal any detectable diffraction peaks corresponding to copper oxide phases. The XRD pattern of Cu_*x*_O/TiO_2_ samples with the copper content of 1.19, 2.28, 4.40, and 4.85 and wt% (Fig. S4†) did not show a distinguishable difference with the XRD pattern of P25 TiO_2_ nanopowders, suggesting highly dispersed and small Cu_*x*_O clusters deposited on TiO_2_. This observation is consistent with our observations using TEM imaging, demonstrating highly dispersed and relatively small Cu_*x*_O clusters over TiO_2_ with an average particle size smaller than 2.7 nm.

**Fig. 3:**
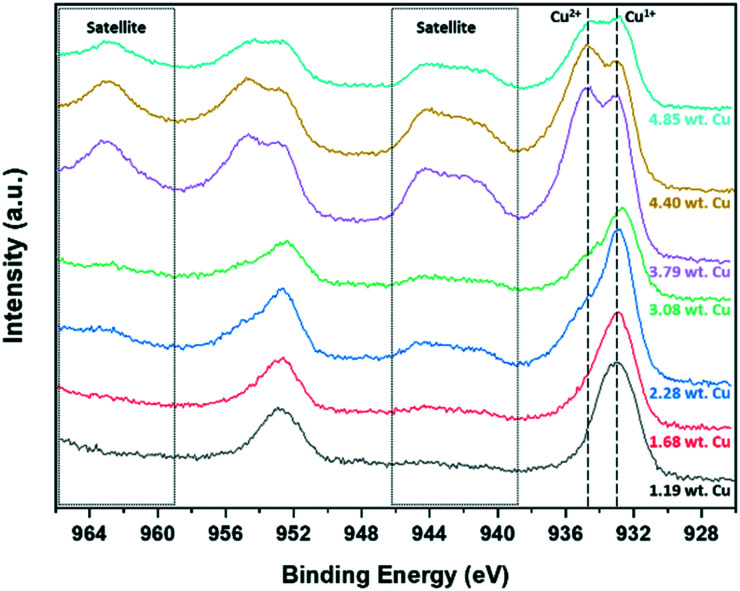
The copper 2p spectra of the fresh ALD synthesized Cu_*x*_O/TiO_2_ samples.

The surface chemistry of the samples and the changes of P25 support upon Cu_*x*_O ALD were studied using XPS analysis. Since the average Cu_*x*_O particle size observed using TEM images for all studied samples is below 4 nm (Fig. 2), the XPS spectra acquired for copper can be assumed as the representative of entire Cu_*x*_O clusters, and it could be employed for describing the bulk of Cu_*x*_O clusters. Fig. 3 shows the high-resolution copper 2p spectra obtained for ALD synthesized Cu_*x*_O/TiO_2_ samples. The different copper species reveal 2p spectra with ∼19.75 eV spin–orbit splitting, which the 2p_3/2_ peak is commonly used for quantification. The metallic copper (Cu^0^) and Cu^1+^ in Cu_2_O reveal a nearby 2p_3/2_ peak at binding energies of 932.6 eV and 932.4 eV for Cu^0^ and Cu^1+^, respectively, which makes the distinction of these two species difficult using XPS. The use of LMM Auger peak is a more efficient solution for identification/quantification of Cu^0^ and Cu^1+^; however, due to overlap of this spectral region with titanium 1s binding energy, we cannot employ LMM Auger spectra for this purpose.^51^ Considering the average size of the ALD synthesized Cu_*x*_O clusters (∼2 nm or smaller) and the oxidative condition of the synthesis process, it is reasonable to assume that the deposited copper is oxidized to some degree and the ALD prepared samples are Cu^0^ free. On the other hand, Cu^2+^ of CuO with a distinct 2p_3/2_ peak at 933.6 eV is easily distinguishable. The Cu^2+^ species also reveal a shake-up satellite peak in the binding energy range of 940–945 eV for 2p_3/2_.^52^ The range that satellite peaks may appear for different copper species is marked in Fig. 3. The copper 2p XPS spectra of Cu_*x*_O/TiO_2_ samples in Fig. 3 indicate that the majority of copper in the sample with low copper content is Cu^1+^, and the Cu^2+^ content becomes clearly detectable in the case of the sample with the copper content of 2.28 wt% or higher, depicting distinguishable satellite peaks and the main 2p_3/2_ peak around 933.6 eV.

The contribution of Cu^1+^ and Cu^2+^ to the Cu 2p_3/2_ spectra was calculated using the method that Biesinger has proposed.^52^ The Cu 2p_3/2_ peak and the corresponding satellite peak were carefully deconvoluted (Fig. S5†); each sample's Cu^1+^ and Cu^2+^ content was calculated by considering the ratio of the area of the main peak of CuO (Cu^2+^) to the shake-up peak area equal to 1.89. The calculated oxidation states enabled us to calculate the average oxidation of copper in the Cu_*x*_O/TiO_2_ samples and consequently approximate the Cu_*x*_O loading of each sample using eqn (S4†); for the details of calculations, please refer to the electronic supporting information. Table 3 summarizes the results of these calculations. These results suggest that the Cu^1+^ is the most likely oxidation state for ultra-fine Cu_*x*_O clusters, and the increase of Cu_*x*_O particle size increases the Cu^2+^ content. As can be seen in Table 3, the increase of copper weight loading, and consequently the Cu_*x*_O particle size, increases the Cu^2+^ content of samples. Accordingly, we can infer that this strong interaction mainly happens at the interface of Cu_*x*_O clusters and TiO_2_, and for the small Cu_*x*_O clusters, it dominates the entire particle, resulting in Cu^1+^ as the only observable copper species. In contrast, for large particles in which the bulk of particle is not entirely affected by the interface, the Cu^2+^ species also became detectable.

**Table 3:** The different copper oxidation states, average copper oxidation state, and approximate Cu_*x*_O loading, calculated using XPS analysis

Sample	Copper loading (wt%)	Cu^1+^ content (%)	Cu^2+^ content (%)	Average copper oxidation state	Calculated CuO_*x*_ loading (wt%)
1	1.19	89.6	13.4	1.13	1.36
2	1.68	74.9	25.1	1.25	1.94
3	2.28	55.0	45.0	1.45	2.7
4	3.08	54.7	45.3	1.45	3.64
5	3.79	8.6	91.4	1.91	4.72
6	4.40	12.0	88.0	1.88	5.44
7	4.85	23.1	76.9	1.77	5.93

**Fig. 4:**
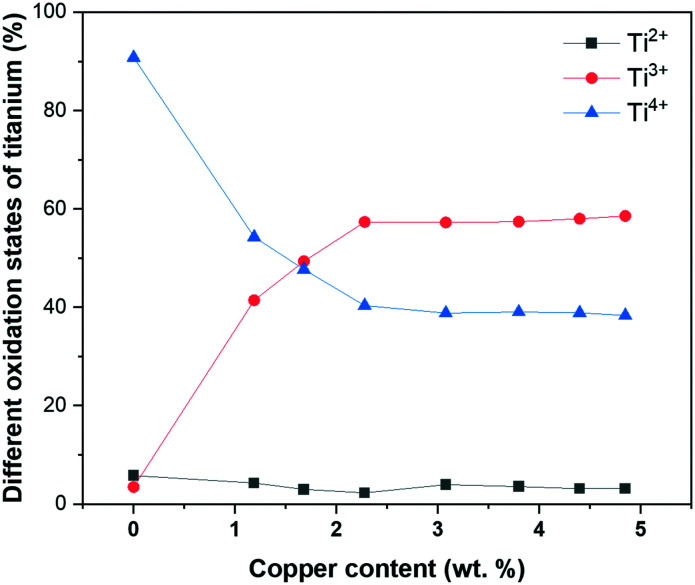
The different titanium oxidation states as a function of copper weight loading in fresh Cu_*x*_O/TiO_2_ samples.

The quantification of the high-resolution titanium 2p spectra of pristine P25 TiO_2_ nanopowder and the ALD synthesized Cu_*x*_O/TiO_2_ samples depicted intriguing trends in the oxidation state of titanium species (Fig. 4). The different titanium species were identified based on the recent work of Biesinger *et al.*,^52^ and the percentage of each oxidation state was calculated by normalizing the 2p_3/2_ peak area of each species to the peak area of the entire 2p_3/2_ (Fig. S6†). As is shown in Fig. 4, the pristine P25 TiO_2_ powder mainly contains Ti^4+^ (*ca.* 90%); however, the Ti^4+^ content decreases drastically upon ALD of Cu_*x*_O onto TiO_2_, and consequently, the Ti^3+^ increases. Whilst the Ti^2+^ content seems to be independent of copper loading and remains constant. The decrease of Ti^4+^ and increase of Ti^3+^ plateaus at copper content higher than 2.28 wt%.

The drastic change of Ti^4+^ and Ti^3+^ content after ALD of Cu_*x*_O clusters indicates the surface modification of P25 during ALD of Cu_*x*_O and a strong interaction between the titanium oxide and copper oxide phases in these samples. This interaction facilitates charge transfer between Cu_*x*_O and TiO_2_,^15,20,53,54^ leading to Ti^4+^ reduction to Ti^3+^; it also stabilizes the Cu_2_O species on TiO_2_.^53–55^ Recently, Huang and co-workers inferred for an incipient wetness impregnation synthesized CuO_*x*_–TiO_2_ system that this strong interaction increases the dispersion of Cu_2_O, consequently the intensity of Cu_2_O–TiO_2_ heterojunction, which is active in photocatalytic hydrogen production.^15^ It is also reported that the formation of Ti^3+^ efficiently hampers the recombination of photogenerated electrons/holes.^56^ Considering the nature of the ALD synthesis, which proceeds *via* chemisorption of precursors onto the substrate, a stronger interaction between the ALD deposited copper add atoms and TiO_2_ substrate is expected than the conventionally impregnated CuO_*x*_ NPs; the chemisorption of ALD precursor on the surface and its dissociation can modify the P25 surface. The surface study of the ALD synthesized Cu_*x*_O/TiO_2_ samples (Fig. 4 and S6†) demonstrates such surface modification and strong interaction, suggesting that the outmost layer of TiO_2_ particles is dominated with Ti^3+^ species due to the strong interaction of highly dispersed ALD deposited Cu_*x*_O clusters and TiO_2_.

### First principles density functional theory simulations of cu/Cu_*x*_O–TiO_2_

**Fig. 5:**
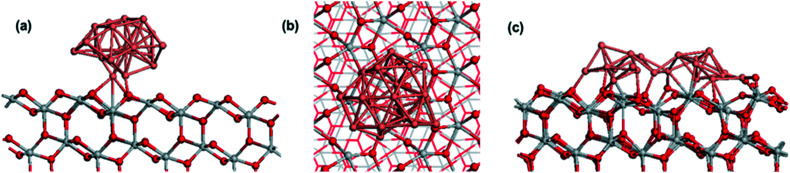
(a) and (b): Side and top views of the atomic structure of Cu_20_ nanocluster adsorbed at anatase (101), (c): relaxed atomic structure of two initially separated Cu_10_ nanoclusters on anatase (101). Color coding: grey spheres represent Ti, red spheres represent O and pink spheres represent Cu.

In addition to XPS analysis, DFT simulations were used to understand better the interactions and oxidation states of the Cu_*x*_O/TiO_2_ system. A Cu_20_ cluster deposited on the anatase (101) surface was the first studied case. Fig. 5(a) and (b) show the atomic structure of this system. The formation of new Cu–O bonds between Cu and surface oxygen can be seen, with Cu–O distances of 1.82, 1.97, and 2.10 Å. The energy gain when the Cu nanocluster adsorbs on the anatase (101) surface is −5.13 eV, indicating a very strong interaction at the TiO_2_ support. Examining oxidation states, two surface Ti cations are reduced to Ti^3+^, with computed Bader charges of 1.72 electrons (compared to 1.32 electrons for Ti^4+^ cations). As a result, two Cu atoms are oxidized to Cu^+^, with computed Bader charges of 16.6 and 16.7 electrons, compared to 17 for metallic copper.

Given the preference for copper clusters to aggregate into larger species with increased coverage, the Cu_20_ nanocluster is compared with two initially separated Cu_10_ nanoclusters on the same anatase (101) surface, and the final structure is shown in Fig. 5-c. After relaxation, the two clusters have aggregated, with the clear formation of Cu–Cu bonds between the two clusters, even for a 0 K relaxation. The oxidation states of the metal cations show 11 reduced Ti^3+^ cations, with computed Bader charges between 1.56 and 2.0 electrons. Copper atoms that directly bind to the anatase surface are oxidized to Cu^2+^ and Cu^+^, with computed Bader charges between 16.2 and 16.4 electrons for Cu^2+^ and 16.5 electrons for Cu^+^.

This indicates that for pure copper metal aggregation into larger clusters will be promoted, reducing dispersion while Cu^2+^, Cu^+^, and Cu^0^ oxidation states will be present. This is not consistent with the finding of Cu^1+^ and Cu^2+^ oxidation states dominating after ALD, and therefore, oxidized Cu nanoclusters interfaced with anatase (101) are studied.

**Fig. 6:**
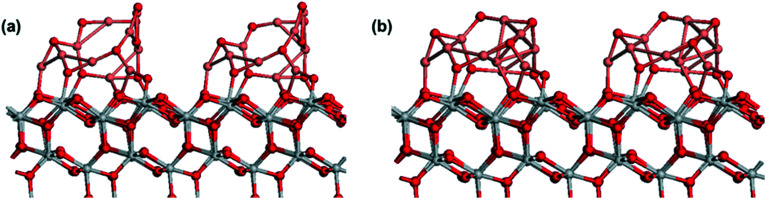
Atomic structure of two Cu_*x*_O nanoclusters adsorbed on anatase (101). (a): Cu_10_O_9_ (b): Cu_10_O_7_ stoichiometries. Color coding: grey spheres represent Ti, red spheres represent O and pink spheres represent Cu.

Fig. 6 shows the atomic structure of two CuO-derived nanoclusters supported on anatase (101). Similar to Cu-anatase (101), two separated CuO nanoclusters were relaxed, and after relaxation, the nanoclusters do not aggregate, which is in contrast to pure copper nanoclusters. This is however, in agreement with the observation of stable Cu_*x*_O particle sizes for a wide range of copper contents (Fig. 2).

In exploring the chemical state of adsorbed CuO, the removal of oxygen (one from each cluster), was investigated and the loss of the first oxygen vacancy (Fig. 6-a) is favorable with a negative energy change of −0.2 eV. Although the energy cost to form the next oxygen vacancy is positive, it is only +0.06 eV, which suggests this will form. The third O vacancy, Fig. 6-b has an energy cost of +1.24 eV per O vacancy, so this is the reduced state of CuO_*x*_-modified TiO_2_, with stoichiometry Cu_10_O_7_.

Examining the oxidation states, partially reduced Ti^3+^ cations, with computed Bader charges of 1.40 electrons, are present. In supported CuO_*x*_, both Cu^1+^ and Cu^2+^ are present, with Bader charges of 10.2–10.3 for Cu^2+^ and 10.5 for Cu^+^. These results agree with the experimental observations from XPS analysis, confirming the TiO_2_ surface modification upon Cu_*x*_O ALD and the strong interaction of Cu_*x*_O clusters with TiO_2_, leading to Ti^4+^ reduction to Ti^3+^.

### Solar hydrogen production

**Fig. 7:**
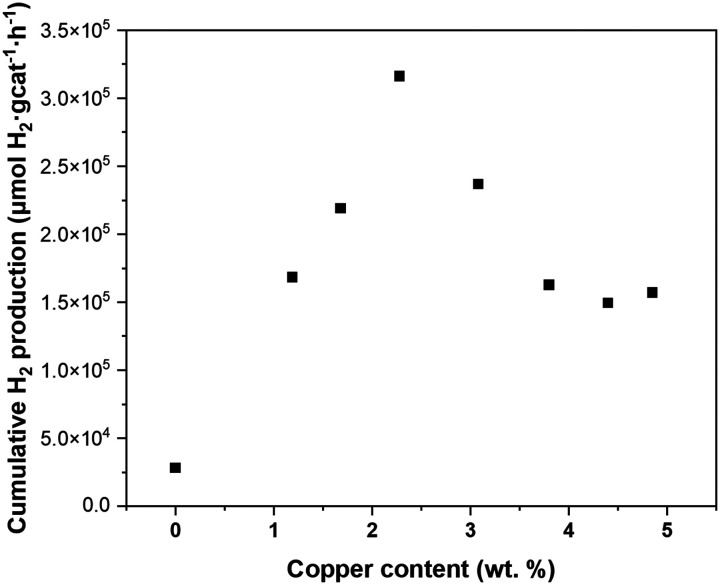
The cumulative hydrogen production after 20 hours of reaction as a function of copper loading in Cu_*x*_O/TiO_2_ photocatalysts.

The solar hydrogen productivity of ALD synthesized Cu_*x*_O/TiO_2_ samples were evaluated and compared with pristine P25 TiO_2_ nanopowder (Fig. 7 and S7†). As Fig. 7 shows, the Cu_*x*_O ALD significantly increases the hydrogen activity of photocatalysts by one order of magnitude compared to pristine P25 TiO_2_, with about 11 times more hydrogen production for the sample with 2.28 wt% copper. Such remarkable activity improvement can be attributed to the co-existence of Cu^1+^ and Ti^3+^ species in Cu_*x*_O/TiO_2_ samples, demonstrated using XPS analysis. The band edge potentials of Cu^1+^ and Cu^2+^ are sufficiently reductive to drive the HER reaction;^2^ additionally, both Cu^1+^ and Cu^2+^ are narrow bandgap p-type semiconductors with a bandgap of 1.7 eV and 2.1 eV, respectively.^57^ Copper(i/ii) oxide deposition on TiO_2_ can narrow the bandgap of the Cu_*x*_O–TiO_2_ system and increase the sunlight utilization, resulting in enhanced photocatalytic activity. Valero *et al.* suggested that the highly dispersed Cu^2+^ species are directly related to high hydrogen productivity.^22^ Furthermore, it is demonstrated that the CuO_*x*_–TiO_2_ undergoes an *in situ* reduction/restructuring under solar radiation, resulting in Cu_2_O–TiO_2_ formation, revealing the Cu_2_O is the active copper species in CuO_*x*_–TiO_2_ photocatalysts.^3,16,17,21,22,58^ The Cu^2+^ species usually reduce to Cu^1+^ or Cu^0^ under light radiation, acting as the effective cocatalyst for the water reduction reaction.

The interfacial interaction between the Cu_*x*_O (p-type semiconductor) with TiO_2_ (n-type semiconductor) forms a heterojunction (Schottky junction) which results in charge transfer and separation between Cu_*x*_O and TiO_2_.^15,19,20,53,54,59^ Such strong interaction and charge transfer can reduce Ti^4+^ to Ti^3+^, which is demonstrated using XPS analysis (Fig. 4) and DFT simulations. It is suggested that the formation of surface Ti^3+^ species has a great contribution to the photocatalytic activity of Cu_2_O–TiO_2_ photocatalyst;^56^ Ji *et al.*^60^ suggested that the surface Ti^3+^ species significantly promote the H-free radical production, which is responsible for enhancing hydrogen production. It is also demonstrated that Ti^3+^ extends the photocatalytic activity of TiO_2_ from UV to visible light, increasing its hydrogen generation rate.^12–14^ This suggests that the drastic increase of Ti^3+^ content on the Cu_*x*_O/TiO_2_ samples surface is the second promotional effect of ALD of Cu_*x*_O clusters on hydrogen production in addition to the known effect of CuO_*x*_ clusters: the increased visible light absorption, and charge carrier separation.^3^ Accordingly, we infer that the ALD grown Cu_*x*_O clusters on TiO_2_ have a dual promotional effect on photocatalytic hydrogen production. The copper content that the highest hydrogen productivity is observed (Fig. 7) coincides with the copper content at which the Ti^3+^ content plateaus (Fig. 4). This unveils a better picture of the relation between the surface Ti^3+^ content and the hydrogen productivity of Cu_*x*_O/TiO_2_ photocatalysts.

### Modified expanding photocatalytic area and overlap model

The characterization of the ALD synthesized Cu_*x*_O/TiO_2_ photocatalysts unveils the dual promotional effect of ALD deposited Cu_*x*_O clusters; furthermore, it reveals the characteristics which are previously identified as the properties of a photocatalyst that is appropriately promoted with copper. These characteristics include highly dispersed Cu_*x*_O particles with a significant amount of Cu^1+^ content and a considerable amount of Ti^3+^ content due to the strong interaction of ALD grown Cu_*x*_O clusters and TiO_2_. Such properties resulted in a highly active photocatalyst for HER reaction, with a minimum of 5 times more hydrogen production than pristine P25 TiO_2_ for Cu_*x*_O/TiO_2_ samples (Fig. 7 and S7†). Beyond the significantly increased solar hydrogen production of Cu_*x*_O/TiO_2_ samples, an intriguing trend in hydrogen productivity of ALD synthesized Cu_*x*_O/TiO_2_ photocatalysts is observable. Fig. 7 indicates a linear increase of hydrogen productivity by increasing copper weight percent until a pinnacle at a copper content of 2.28 wt%, then it declines and plateaus afterward. On the other hand, the surface Ti^3+^ content of these samples increases with almost a linear trend and plateaus at the copper content of 2.28 wt% (Fig. 4). Furthermore, the PSD of Cu_*x*_O clusters becomes wider by the increase of copper content (Fig. S3†), and their particle size starts growing at copper content higher than 4.40 wt% (Fig. 2). This suggests a direct relation between the photo catalytic activity of Cu_*x*_O/TiO_2_ photocatalyst and its Ti^3+^ content, copper oxide loading, and copper oxide particle size. We modified the EPAO developed by Mills *et al.*^29^ to quantify this relation.

The EPAO model^29^ is a kinetic model based on the previous metal support interface (MSI) model of Bowker *et al.*^30^ The EPAO model describes the relationship between the cocatalyst weight loading and hydrogen production rate during photocatalytic methanol reforming using six main assumptions. These assumptions are described in detail in the original work.^29^ The model assumes that the metal particles deposited on TiO_2_ support (for a Pt/TiO_2_ system) form an electric field with the surrounding TiO_2_, resulting in a photocatalytically active circular area around the particle whose radius is a simple linear function of the radius of the metal particle. They also assumed that the number of metal particles is constant, and their size grows by increasing metal loading. Based on the insight obtained into our ALD synthesized Cu_*x*_O/TiO_2_ photocatalysts, we modified the original EPAO model *via* more realistic assumptions and adapted it with our observations; however, the main assumptions of this model are maintained. These new assumptions of the modified EPAO (M-EPAO) model are based on the characteristics of the ALD synthesized Cu_*x*_O/TiO_2_ samples, making it a more realistic model; the four new assumptions are as below:

i. The number of cocatalyst islands depends on the cocatalyst loading and the dominant stage of the nucleation and growth processes. During the nucleation stage, the number of islands increases, while when the growth becomes dominant, the number of the particles may remain constant or decrease, depending on the governing mechanism.

ii. The average size of cocatalyst islands remains constant during the nucleation stage, and it increases during the growth stage.

iii. The increase of hydrogen production rate (*r*(H_2_)) stems from the rise of the number of cocatalyst islands in the cocatalyst loading range in which the nucleation is dominant, leading to an increase of photocatalytically promoted area.

iv. The heterojunction of cocatalyst/support (p–n junction in our case) and their strong interaction leads to the formation of a depletion zone (area) around the cocatalyst particles. As long as these areas do not overlap much, their extension increases the hydrogen production rate (*r*(H_2_)).

**Fig. 8:**
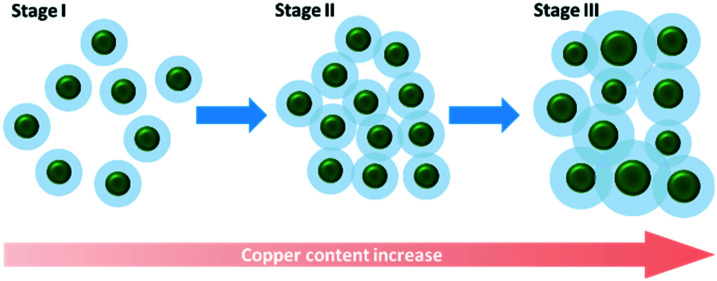
The schematic representation of three stages in the M-EPAO model.

Like the original EPAO model, the M-EPAO model assumes that each cocatalyst particle creates a photocatalytically promoted area (PPA) around it (a depletion zone), which its radius is a linear function of the radius of the cocatalyst particle. As Fig. 8 illustrates, the M-EPAO model consists of three stages. In the first stages, due to the low population of the cocatalyst particles on the surface, the distance between the particles is long, and the PPA does not overlap. At this stage, the increase of copper oxide loading and consequently growth of new cocatalyst clusters on the surface increases the PPA, resulting in increased hydrogen production. The photocatalyst activity will increase by the increase of cocatalyst content until the point that the PPA of individual clusters starts overlapping due to the increasing number of particles (near the maximum activity copper content). This stage describes the ascending part of Fig. 7.

The overpopulation of cocatalyst clusters and overlap of PPA marks the decay of activity of the photocatalyst as a function of the cocatalyst loading. The photocatalytic activity decay resulting from PPA overlap can be attributed to local depletion of the reaction intermediates on the catalyst surface, which has a strong negative effect on the reaction rate in the case of densely spaced surface islands.^61^ The overlap of PPA also results in interference of the electrical field around the cocatalyst particles and reduces the electron–hole separation efficacy.^29,59^ The negative effect of PPA overlap outweighs the promotional effects of the cocatalyst, and further copper added to the photocatalyst does not contribute to activity enhancement but leads to hydrogen production rate decay. This situation is described in stage II of the M-EPAO model, representing the descending section of Fig. 7. The last stage of the M-EPAO model deals with the cocatalyst loading, which above it the particle growth dominates. At this stage, due to the coalescence of some of the particles, the number of cocatalyst particles decreases; nevertheless, as a result of the growth of cocatalyst particles and reduction in their number, the amount of available PPA and the overlapped area do not change significantly, and the photocatalytic activity of samples does not change considerably. The last stage of the M-EPAO model describes the range of cocatalyst loading in which the hydrogen productivity remains constant (copper wt% higher than 3.79 in Fig. 7).

Since the M-EPAO model needs the Cu_*x*_O particle size to calculate the PPA, we used the Cu_*x*_O content approximated using XPS analysis (Table 3) in our calculation. The Cu_*x*_O particle size as a function of Cu_*x*_O content was approximated using a linear function (Fig. S8-a†), including two stage s of nucleation (constant size) and particle diffusion and coalescence (growth). This function is used in the M-EPAO model to estimate the Cu_*x*_O particle size in different Cu_*x*_O content. Also, the number of Cu_*x*_O clusters was calculated using eqn (S9†), and a curve was fitted to this data (Fig. S8-b†). This figure shows how the population of Cu_*x*_O particles on the surface is changing.

**Fig. 9:**
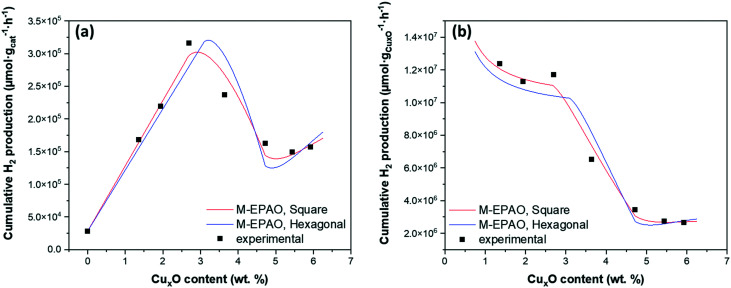
The cumulative hydrogen production after 20 hours of reaction normalized by the mass of (a) photocatalyst and (b) Cu_*x*_O as a function of Cu_*x*_O loading. The solid lines are generated using the M-EPAO model for square (black) and hexagonal (blue) packing of Cu_*x*_O clusters.

We considered two surface packings of square and hexagonal for Cu_*x*_O cluster arrangement on the Cu_*x*_O/TiO_2_ particles. By implementing the particle growth function in the M-EPAO model and optimizing its variables (*a* and *b*′ in eqn (S7†)) in Matlab *via* minimizing the sum of square errors using the Global search function, the M-EPAO model fit to the experimental data was obtained and is plotted in Fig. 9 (for the details of calculations, please refer to the ESI;† also, the Matlab code is provided). The hydrogen production rate normalized to the mass of photocatalyst (μmol g_cat_^−1^ h^−1^) and the mass of Cu_*x*_O in the photocatalyst (μmol g_Cu_*x*_O_^−1^ h^−1^), predicted using the M-EPAO models, are compared to the experimental values in Fig. 9. A quick visual comparison indicates that the M-EPAO model is able to predict the hydrogen production rate pretty well, especially for the square packing of Cu_*x*_O clusters. The model using the square packing of Cu_*x*_O clusters also shows smooth inflection near the maximum and minimum H_2_ productivity points, making its predictions closer to the experimental data. The average absolute relative deviations (AARD) calculated for square and hexagonal packing are 4.8% and 10.1%, respectively, demonstrating that the M-EPAO model fits better with the experimental data using square packing of Cu_*x*_O clusters. However, we should emphasize that due to the random arrangement of Cu_*x*_O clusters on the surface of the photocatalyst, neither square nor hexagonal packings cannot correctly describe the arrangement of Cu_*x*_O clusters. Therefore, the predicted H_2_ productivity rate using the M-EPAO model with the current assumptions for the dispersion of the cocatalyst particles on the surface will deviate from the experimental data to some degree; a more detailed model, taking the surface growth mechanisms into account and providing a better picture of the dispersion of Cu_*x*_O clusters on the surface, may result in more accurate predictions.

**Fig. 10:**
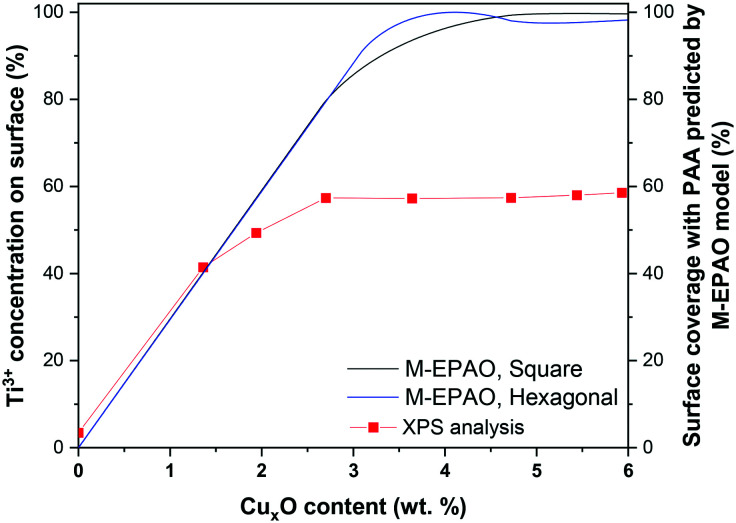
The Ti^3+^ content on the photocatalyst surface obtained with XPS analysis, and surface PPA coverage of photocatalyst as a function of Cu_*x*_O content calculated with the M-EPAO model for square and hexagonal packing of Cu_*x*_O cluster.

The M-EPAO model enables us to calculate the photocatalytically promoted area (*A*_T_ in eqn (S5†)) of the photocatalyst created due to the strong interaction of Cu_*x*_O cocatalyst clusters and TiO_2_ support. Fig. 10 illustrates the surface coverage of Cu_*x*_O/TiO_2_ photocatalyst with PPA as a function of Cu_*x*_O content calculated with M-EPAO model for square and hexagonal Cu_*x*_O cluster packing and compares them with the surface Ti^3+^ content on the photocatalyst measured using XPS analysis. Fig. 10 reveals the similarity between the PPA development on Cu_*x*_O/TiO_2_ photocatalyst (calculated value) and the trend of surface Ti^3+^ increase (experimental data); this demonstrates that the assumptions of the M-EPAO model are valid, and it can consistently describe the cocatalyst decorated photocatalyst systems. It also agrees with the previous observations, suggesting the significant contribution of Ti^3+^ to photocatalytic activity of Cu_*x*_O/TiO_2_ catalysts.^56,60^ The Ti^3+^ species are produced due to the transfer of photo-excited electrons of Cu_*x*_O clusters to TiO_2_,^62^ and the increase of Ti^3+^ content in Cu_*x*_O/TiO_2_ samples indicates the expansion of the depletion zone on the photocatalyst. It can be inferred that the depletion zone around the cocatalyst particles is the photocatalytically active area, and its growth increases the photocatalyst activity. It is worth noting that the maximum surface Ti^3+^ content that we observe using XPS is ∼60%, while the M-EPAO model predicts that at the Cu_*x*_O content above 3.5 wt% the photocatalyst surface is almost covered with the PPA. This discrepancy is likely to arise from two points: 1) the speciation of TiO_2_ in the depletion zone is unknown for us, and we cannot consider it entirely consists of Ti^3+^. Nevertheless, since Ti^3+^ is the main product of the strong interaction of Cu_*x*_O clusters and TiO_2_, we correlate the size of depletion zone to the amount of Ti^3+^. 2) Considering *ca.* 1 keV kinetic energy of Ti 2p photoelectrons (when using aluminum Kα radiation for XPS analysis), about one-third of the detected Ti 2p photoelectrons are coming from the depth of 4 nm or more. This suggests that a considerable part of the detected Ti^4+^ signal might originate from the interior of TiO_2_ NPs, which is not affected by the Cu_*x*_O clusters; however, we cannot exclude the contribution of this portion of Ti^4+^ to the overall Ti 2p XPS signal, while they are not present in the model.

Recently, Mills *et al.*^29,63^ successfully visualized the concept of photocatalytically active area around a platinum dot deposited on TiO_2_*via* soot oxidation experiment. Our XPS analysis, in agreement with their observation, shows how the surface coverage of photocatalyst with Ti^3+^, or in other words the expansion of depletion zone, increases the photocatalytic activity of cocatalyst decorated photocatalysts. Our results suggest that the depletion zone formed around the cocatalyst particles, due to charge separation at the heterojunction, is the main active zone for hydrogen production during photocatalytic H_2_ evolution from a methanol/water mixture. The M-EPAO model can be employed to describe the photocatalyst promoted by surface decoration using metal or metal oxide clusters, and it can predict the optimum cocatalyst loading to maximize the photocatalytic activity *via* maximizing the photocatalytically active area.

## Conclusions

A set of Cu_*x*_O/TiO_2_ photocatalysts with high control over the Cu_*x*_O cluster size was prepared using ALD. TEM imaging and particle size distribution analysis showed a narrow PSD with an average Cu_*x*_O particle size of ∼1.7 nm for the samples with a copper content below 3.79 wt%. The XPS analysis of these samples revealed Cu^1+^ as the dominant copper species for low copper content, while Cu^2+^ increases with higher copper content. The XPS analysis also revealed the drastic effect of Cu_*x*_O ALD on the oxidation state of titanium, depicting a significant reduction of Ti^4+^ reduction to Ti^3+^. The DFT simulations also showed the strong interaction of Cu_*x*_O and TiO_2_, leading to Ti^4+^ reduction to Ti^3+^ and Cu_*x*_O cluster stabilization. The results of photocatalytic hydrogen production and characterization of Cu_*x*_O/TiO_2_ samples unveil the dual promotional effect of ALD grown Cu_*x*_O clusters, *i.e.*, generation of a substantial amount of surface Ti^3+^, and improved charge carrier separation and increased visible light absorption. The copper content at which the hydrogen productivity is maximum coincides with the copper content at which the Ti^3+^ content plateaus. This illustrates the effect of the Ti^3+^ content on the hydrogen productivity of copper-promoted TiO_2_ photocatalysts and enables us to correlate the solar hydrogen production rate to the cocatalyst surface density, weight loading, and size. We modified the EPAO model to describe the behavior of Cu_*x*_O/TiO_2_ system based on Cu_*x*_O content and average particle size. The similar trend of the increase of the surface Ti^3+^ content and the photocatalytically promoted area, calculated with the M-EPAO model, suggests that the depletion zone formed around the Cu_*x*_O clusters is the main active area for hydrogen production, and its expansion increases the hydrogen production rate. However, the overlap of the depletion zones results in activity loss of photocatalyst; hence, the increase of cocatalyst content will enhance the hydrogen rate until the point that the depletion zones of the individual cocatalyst clusters start to overlap.

## Conflicts of interest

There are no known competing financial interests or personal relationships that could have appeared to influence the work reported in this paper.

## Supplementary Material

CY-012-D2CY00400C-s001
