# Supplementary material for: Dual promotional effect of CuxO clusters grown with atomic layer deposition on TiO2 for photocatalytic hydrogen production
Source: Catal Sci Technol. 2022 Jun 8;12(14):4511–23. doi: 10.1039/d2cy00400c (PMC9291445; doi:10.1039/d2cy00400c)
Supplement: CY-012-D2CY00400C-s001 [file CY-012-D2CY00400C-s001.pdf]

Electronic Supplementary Material (ESI) for Catalysis Science & Technology.
This journal is © The Royal Society of Chemistry 2022

Electronic supporting information for

Dual promotional effect of Cu_xO clusters grown with atomic layer deposition on TiO_2 for photocatalytic hydrogen production

Saeed Saedy[*a], Nico Hiemstra[a], Dominik Benz[a], Hao Van Bui[b], Michael Nolan[c], J. Ruud van Ommen[a]

[a] Department of Chemical Engineering, Delft University of Technology, Van der Maasweg 9, 2629 HZ, Delft, The Netherlands

[b] Faculty of Materials Science and Engineering, Phenikaa University, Yen Nghia, Ha-Dong District, Hanoi 12116, Vietnam

[c] Tyndall National Institute, University College Cork, Lee Maltings, Dyke Parade, Cork, T12 R5CP, Ireland

[*]corresponding author: s.saedy@tudelft.nl

The details of XPS analysis and approximation of Cu_xO content

The surface chemistry of the ALD synthesized Cu_xO/TiO_2 samples were studied using the XPS technique. The content of different copper species (Cu^{1+} and Cu^{2+}) was quantified based on the method that Biesinger has proposed [1, 2]. This method uses the shake-up peaks that are present in the spectra of Cu^{2+} but are absent Cu^0 or Cu^{1+} spectra. The shake-up peaks are the result of the interaction of the outgoing photoelectrons with the valance electrons, leading to the excitation of the valance electrons to a higher energy level. As a result of such inelastic interactions, the outgoing core photoelectron loses few electron volts of energy, producing shake-up peaks. **Figure S1** shows the $2p_{3/2}$ spectra of two samples with low and high Cu^{2+} content (1.19 and 3.79 wt. %, respectively). The different copper species, Cu^0, Cu^{1+}, and Cu^{2+}, contribute to the main emission line of copper (region A in **Figure S1**), while the shake-up satellite peaks (region B in **Figure S1**) stem from Cu^{2+}. Accordingly, the shake-up satellite peaks of region B can be assumed as the fingerprint of Cu^{2+}, and its absence indicates the presence of Cu^0/Cu^{1+} only. Biesinger suggests that the content of different copper species should be calculated by taking the signal of the main emission line and the shake-up peaks.

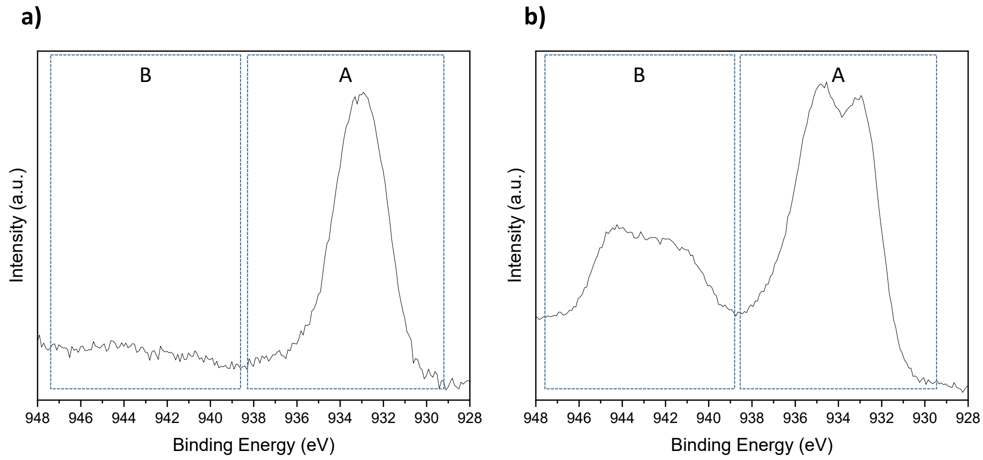

Figure S1. The Cu $2p_{3/2}$ spectra of Cu_xO/TiO_2 sample with a copper content of 1.18 wt. % (a) and 3.79 wt. % (b).

It is worth noting that the metallic copper (Cu^0) and Cu^{1+} in Cu_2O show a very close $2p_{3/2}$ peak at binding energies of 932.6 eV and 932.4 eV for Cu^0 and Cu^{1+}, respectably. The distinction of these two species is challenging using XPS; however, they can be clearly distinguished using the LMM Auger peak. Since in our samples, the main matrix is TiO_2, and this spectral region overlaps with Ti 1s, we cannot employ Auger spectroscopy for this purpose [3]. Also, since the size of the ALD synthesized Cu_xO clusters observed using

TEM imaging is ~2 nm or smaller, and the synthesis process had an oxidative atmosphere at 250°C, we assumed that the ALD deposited copper is oxidized to some degree, and the Cu_xO/TiO_2 samples are Cu^0 free.

Biesinger [1, 2] has proposed the following equations to calculate the relative concentration of Cu^{1+} and Cu^{2+} on the surface of a copper-containing sample:

$$\%Cu^{1+} = \frac{A2}{A+B} \times 100 = \frac{A - A1}{A+B} \times 100 = \frac{A - \left(A1_s/B_s\right)B}{A+B} \times 100$$ (**Equation S1**)

$$\%Cu^{2+} = \frac{B + A_1}{A+B} \times 100 = \frac{B\left(1 + \left(A1_s/B_s\right)\right)}{A+B} \times 100$$ (**Equation S2**)

where A1 is the peak area of the main signal of Cu^{2+}, A2 is the peak area of the main signal of Cu^{1+}, and B is the peak area of shake-up satellite peaks. The accuracy of these two equations depends on the accurate determination of the ratio between the main peak/shake-up peak areas ($A1_s/B_s$) for a 100% pure Cu^{2+} sample. We used 1.89 for the $A1_s/B_s$, reported for Cu^{2+} by Biesinger for our calculations [1]. The relative concentrations of Cu^{1+} and Cu^{2+} in Cu_xO/TiO_2 samples were calculated using equations **S1** and **S2**, and the results are summarized in **Table 2** of the main text.

Using the calculated values for Cu^{1+} and Cu^{2+} content in ALD synthesized Cu_xO/TiO_2 samples, the average oxidation state of copper is calculated:

$$Cu_{avg}^{ox} = \frac{\%Cu^{1+} + 2 \times \%Cu^{2+}}{100}$$ (**Equation S3**)

Using the average oxidation state of copper and assuming a stoichiometric ratio between copper and oxygen, the weight loading of Cu_xO in the ALD synthesized Cu_xO/TiO_2 samples were calculated:

$$w_{Cu_xO}\% = w_{Cu}\% * \left(1 + \frac{Cu_{avg}^{ox}}{O^{ox}} * \frac{M_{w,\,O}}{M_{w,\,Cu}}\right)$$ (**Equation S4**)

where $M_{w,\,O}$ is the molar mass of oxygen assumed to be 15.999 g·mol^{-1} and $M_{w,\,Cu}$ is the molar mass of the copper assumed to be 63.546 g·mol^{-1}. The oxidation state of oxygen is assumed to equal 2. The calculated weight loadings of ALD synthesized Cu_xO/TiO_2 samples are presented in **Table 2** of the main text.

Formulations and parameters of Modified expanding photocatalytic area and overlap model

The Modified expanding photocatalytic area and overlap (M-EPAO) model uses similar formulations that the original EPAO model uses [4], and the equations are modified to satisfy the assumption/condition of the M-EPAO model. In addition to the main difference between the M-EPAO model and the original EPAO model described in the main text, the M-EPAO model takes the photoactivity of pristine P25 TiO_2 into account. In contrast, the original EPAO model has associated the rate of hydrogen generation with the total photocatalytically promoted area, A_T [4]. During stages I and II of the M-EPAO model, the number of cocatalyst clusters is increasing, then during stage III, the clusters start growing.

The parameters used in the M-EPAO model are listed in **Table S1**. These parameters are obtained in different ways. Some parameters are defined using the experimental data, and others are calculated. The parameters *a*, *b'*, and *k* are obtained via fitting the model to the experimental data via minimizing the sum of square errors using the Globalsearch function in Matlab. Calculating these parameters directly is difficult or not possible, and for that reason, they are optimized.

As explained in the main text, the M-EPAO model is divided into three different sections. The first section is defined as the system before the theoretical ideal weight loading; at this point, there is no photocatalytically promoted area (PPA, A_T) overlap. The second section of the model is defined as the system after the ideal

weight loading where the PPA overlap occurs. The third section of the model is characterized by the increase of the cocatalyst clusters size. The moment when certain sections of the model are in effect is dependent on the nucleation and growth behavior. The base equation of the M-EPAO model is the hydrogen production rate:

$$r(H_2) = (k * A_T + k_{base} * A_{base}) * SA \qquad \textbf{(Equation S5)}$$

Table S1. The parameters used in the M-EPAO model.

Parameter	unit	definition	Source
**w%**	%	the weight percentage of cocatalyst on the surface	experimental/model
$r(H_2)$	$umol\cdot h^{-1}\cdot g_{catalyst}^{-1}$	hydrogen production per gram catalyst	experimental/model
k	$umol\cdot h^{-1}\cdot m^{-2}_{active}$	the activity of the perimeter	optimized
k_{base}	$umol\cdot h^{-1}\cdot m^{-2}_{active}$	base activity of TiO_2	experimental
A_T	$m^2_{active}\cdot m^{-2}_{catalyst}$	photocatalytically promoted area per TiO_2 area	calculated
A_{base}	$m^2_{active}\cdot m^{-2}_{catalyst}$	unpromoted area per area TiO_2	calculated
N	$sites\cdot m^{-2}_{catalyst}$	number of cocatalyst clusters per area of TiO_2	calculated
r	m	the radius of cocatalyst cluster	experimental/interpolated
r_z	m	the radius of the photocatalytically active area	calculated
a	-	the linear constant of photocatalytically active area growth	optimized
b'	-	the linear constant of photocatalytically active area change due to cluster size growth	optimized
R	m	cocatalyst cluster interparticle distance	calculated
r^*	m	constant cluster size at low weight loading	experimental
f	-	packing parameter	assumption
ΔA	m^2	overlapping deactivated area	calculated
c	$nm\cdot w\%^{-1}$	the cocatalyst growth constant	calculated
SA	$m^2_{catalyst}\cdot g_{catalyst}^{-1}$	specific surface area	material property
ρ	$g\cdot m^{-3}$	the mass density of cocatalyst	material property

Equation S5 considers the activity of the PPA (A_T) and the unpromoted area of P25 TiO_2 (pristine area - A_{base}). It calculates the rate of hydrogen production based on a rate constant of k and k_{base} per area of photocatalytically promoted and the unpromoted area, respectively. The specific surface area of P25 TiO_2 (SA) is employed to change the unit of the rate from $\mu mol_{H_2} \cdot m_{cat}^{-2} \cdot h^{-1}$ to $\mu mol_{H_2} \cdot g_{cat}^{-1} \cdot h^{-1}$.

The first stage of the M-EPAO model deals with the situation that the increase of the number of cocatalyst clusters results in the rise in A_T and consequently increases the hydrogen production rate. The first section extends to the point where the maximum surface coverage with PPA occurs. At this point, the increase of Cu_xO contents causes PPA overlap leading to the deactivation of the photocatalyst. In this situation, the growth of overlapped area slows down the hydrogen production rate, and after a pinnacle, the activity suppressing effect of PPA overlap becomes dominant, and the second stage of the M-EPAO model begins. At this point, the copper or Cu_xO weight loading is optimum concerning the highest hydrogen production rate. When PPA overlap begins, the interparticle distance of Cu_xO clusters (R) is double PPA radius (r_z); this indicates that the photocatalytically promoted area surrounding the individual Cu_xO clusters touch but do not overlap. Then, the further copper deposited on P25 Ti_2O leads to new Cu_xO cluster formation and, consequently, PPA overlap. The PPA overlap initiates photocatalytic activity loss. In the third stage, the Cu_xO clusters start to grow due to particle diffusion and coalescence, impacting the surface density of clusters and the available PPA in the model. For stages I and II which nucleation is the dominant mechanism, a fixed Cu_xO cluster size is defined (r^*) is determined. During stage III, the radius of Cu_xO clusters is assumed to grow linearly by the increase of Cu_xO content (**Figure S5-a**). The growth Cu_xO constant (c) was obtained using linear fitting of the particle size measured using TEM images as a function of Cu_xO content.

The photocatalytically active area around Cu_xO clusters is calculated using parameters *a* and *b'*. The parameter *a* defines the active area based on the initial constant surface island size. As the surface islands' size changes, the size of the PPA will change as well. This change in PPA size is due to a change of surface islands' size and is defined using the *b'* parameter. The radius of photocatalytically active area around Cu_xO cocatalyst clusters (r_z) is calculated using equations below for three stages of the M-EPAO model:

Stage I and III:

$$r_z = a \times r^* \qquad \textbf{(Equation S6)}$$

Stage III:

$$r_z = a \times r^* + b^{'}(r - r^*) \qquad \textbf{(Equation S7)}$$

where

$$r = 0.5 \times 10^{-9} \times \left(2r^* \times 10^{-9} + (w\% - w\%_{growth}) \times c\right) \qquad \textbf{(Equation S8)}$$

Equation S8 predicts the size increase of Cu_xO clusters as a function of Cu_xO content by taking the content at which the cluster growth begins into account. The constant c can be obtained via linear curve fitting the experimental data.

The interparticle distance (R) of Cu_xO clusters depends on surface density (N) and the packing of clusters on the surface. The surface density of Cu_xO clusters can be calculated using the equation below:

$$N = \frac{w_{Cu_xO}\%}{100 \cdot SA \cdot \rho \frac{2\pi r^3}{3}} \qquad \textbf{(Equation S9)}$$

For hexagonal and square packing, the interparticle distance can be calculated using equations **S9** and **S10**, respectively:

$$R = \sqrt{\frac{2}{\sqrt{3}N}} \qquad \textbf{(Equation S9)}$$

$$R = \sqrt{\frac{1}{N}} \qquad \textbf{(Equation S10)}$$

During stage I, the interparticle distance is large enough to avoid PPA overlap ($R \geq 2r_z$); hence the overlapped area is zero ($\Delta A=0$). Accordingly, A_{base} can be calculated as:

$$A_{base} = 1 - N(\pi r_z^2 + \frac{f}{2}\Delta A) \qquad \textbf{(Equation S11)}$$

where f is the packing factor of Cu_xO clusters on the surface of P25 TiO_2 with the value of 4 and 6 for square and hexagonal packing, respectively.

The highest Cu_xO content in which the hydrogen rate is maximum, the loading/packing of Cu_xO particles is optimum/ideal so that the highest surface coverage with PPA can be achieved. Above this Cu_xO content, the PPA overlap outweighs the promotional effect of Cu_xO clusters. **Figure S2** shows how the PPA overlap is defined in the model.

Figure S2. Overlap of photocatalytically promoted area by the decrease of Cu_xO interparticle distance.

The overlapped area (ΔA) can be calculated using the equation below:

$$\Delta A = r_z^2 \cos^{-1}\left(\frac{d}{r_z}\right) - d\sqrt{r_z^2 - d^2} + r_z^2 \cos^{-1}\left(\frac{d}{r_z}\right) - d_2\sqrt{r_z^2 - d^2}$$

(**Equation S12**)

where $d = \frac{R}{2}$.

Equation S12 is adapted based on the solution provided by Assencio for the intersection area of two circles [5].

By having the overlap area, the photocatalytically promoted area (A_T) can be calculated using **Equation S13**:

$$A_T = N\left(\pi r_z^2 - f\Delta A - \pi r^2\right)$$

(**Equation S13**)

Having the A_T and A_{base}, we can calculate the hydrogen rate using **Equation S5**.

A Matlab code is developed to fit the model using these equations to the experimental data and optimize the three model parameters, i.e., *a*, *b'*, and *k* (the code is provided). The optimized values are summarized in **Table S2**. The M-EPAO model fits well with the experimental data using the values presented in **Table S2**. The model's average absolute relative deviation (AARD) from the experimental data (calculated using **Equation S14**) indicates that the model using square packing of Cu_xO clusters fits better with the experimental data.

$$AARD\ \% = \left[\frac{\sum_{i=1}^{n}\left(\frac{\left|\theta_{E,i} - \theta_{m,i}\right|}{\theta_{E,i}}\right)}{n}\right] \times 100$$

(**Equation S14**)

where $\theta_{E,i}$ is the experimental data, and $\theta_{m,i}$ is the corresponding value obtained from M-EPAO model.

Table S2. The fitting parameters, obtained from the fitting of the M-EPAO model to experimental data using the square and hexagonal pickings.

Cu_xO cluster packing	*k* (μmol·m^{-2}·h^{-1})	*a*	*b'*	AARD (%)
**Square**	9056.5	2.26	3.30	4.8
**Hexagonal**	8610.9	2.24	3.16	10.1

References

[1] M.C. Biesinger, Advanced analysis of copper X-ray photoelectron spectra, Surface and Interface Analysis, 49 (2017) 1325-1334.
[2] M.C. Biesinger, B.R. Hart, R. Polack, B.A. Kobe, R.S.C. Smart, Analysis of mineral surface chemistry in flotation separation using imaging XPS, Minerals Engineering, 20 (2007) 152-162.

[3] P.A. DeSario, J.J. Pietron, T.H. Brintlinger, M. McEntee, J.F. Parker, O. Baturina, R.M. Stroud, D.R. Rolison, Oxidation-stable plasmonic copper nanoparticles in photocatalytic TiO2 nanoarchitectures, Nanoscale, 9 (2017) 11720-11729.
[4] A. Mills, M. Bingham, C. O'Rourke, M. Bowker, Modelled kinetics of the rate of hydrogen evolution as a function of metal catalyst loading in the photocatalysed reforming of methanol by Pt (or Pd)/TiO2, Journal of Photochemistry and Photobiology A: Chemistry, 373 (2019) 122-130.
[5] D. Assencio, The intersection area of two circles, 2017.

Figure S3.

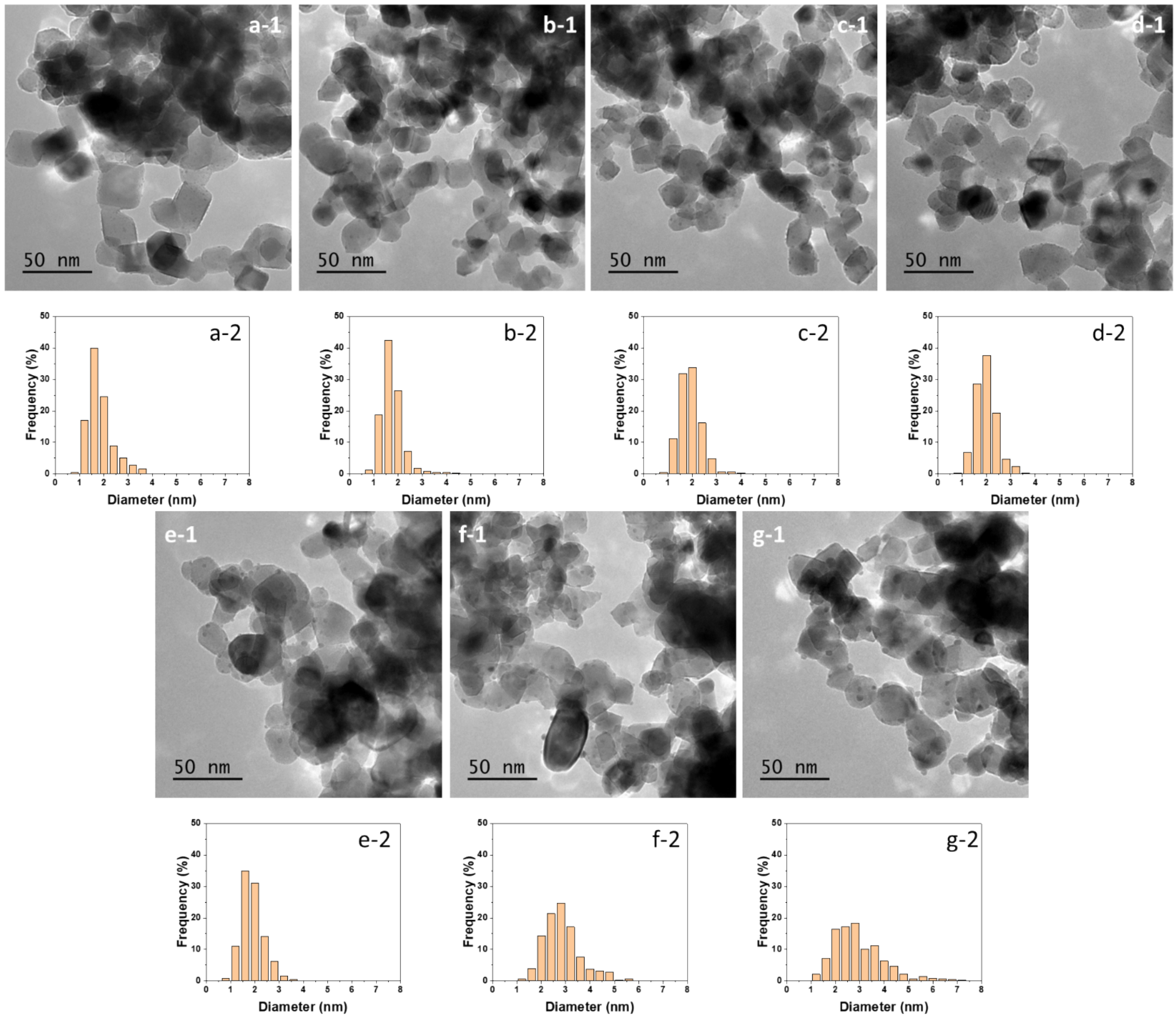

Figure S3. The TEM images and particle size distribution histograms for ALD synthesized Cu_xO/TiO_2 samples with copper content of 1.19 wt. % (a), 1.68 wt. % (b), 2.28 wt. % (c), 3.08 wt. % (d), 3.79 wt. % (e), 4.40 wt. % (f), and 4.85 wt. % (f).

Figure S4.

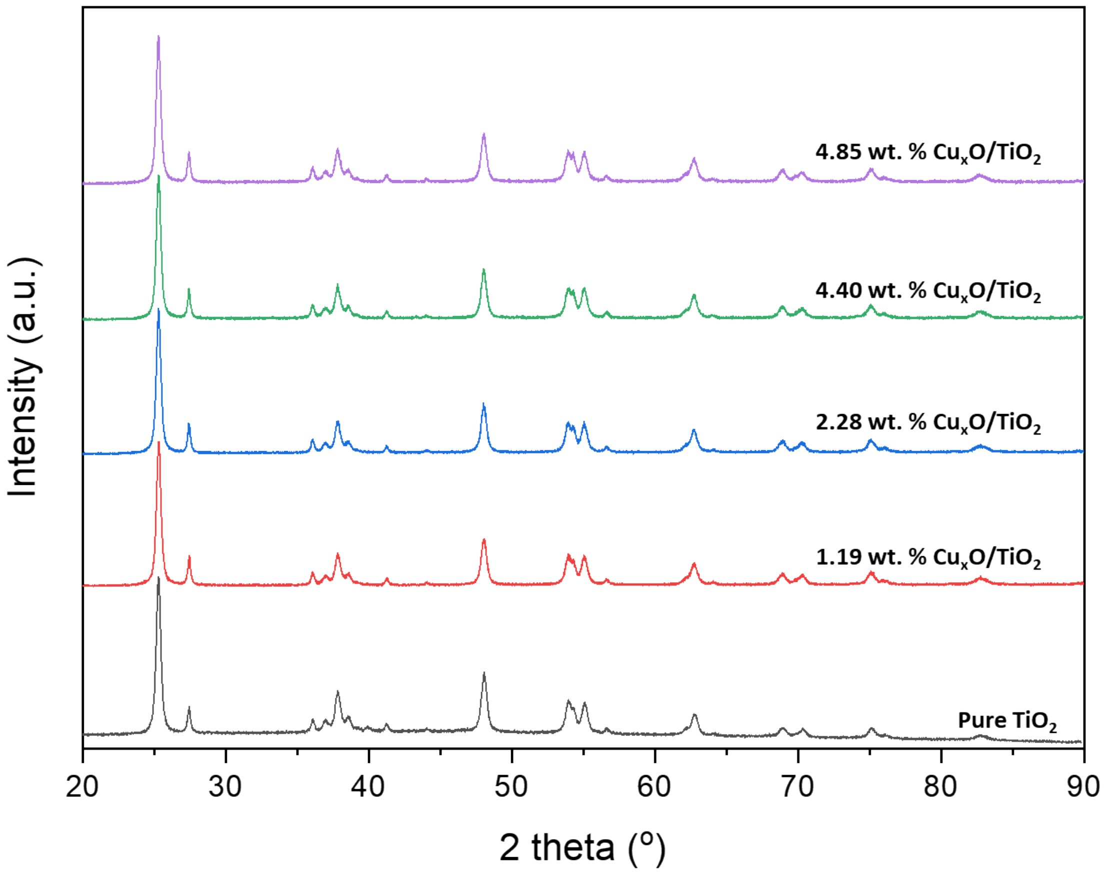

Figure S4. X-ray diffraction patterns of ALD synthesized Cu_xO/TiO_2 samples with copper content of 1.19, 2.28, 4.40, and 4.85 wt. %.

Figure S5.

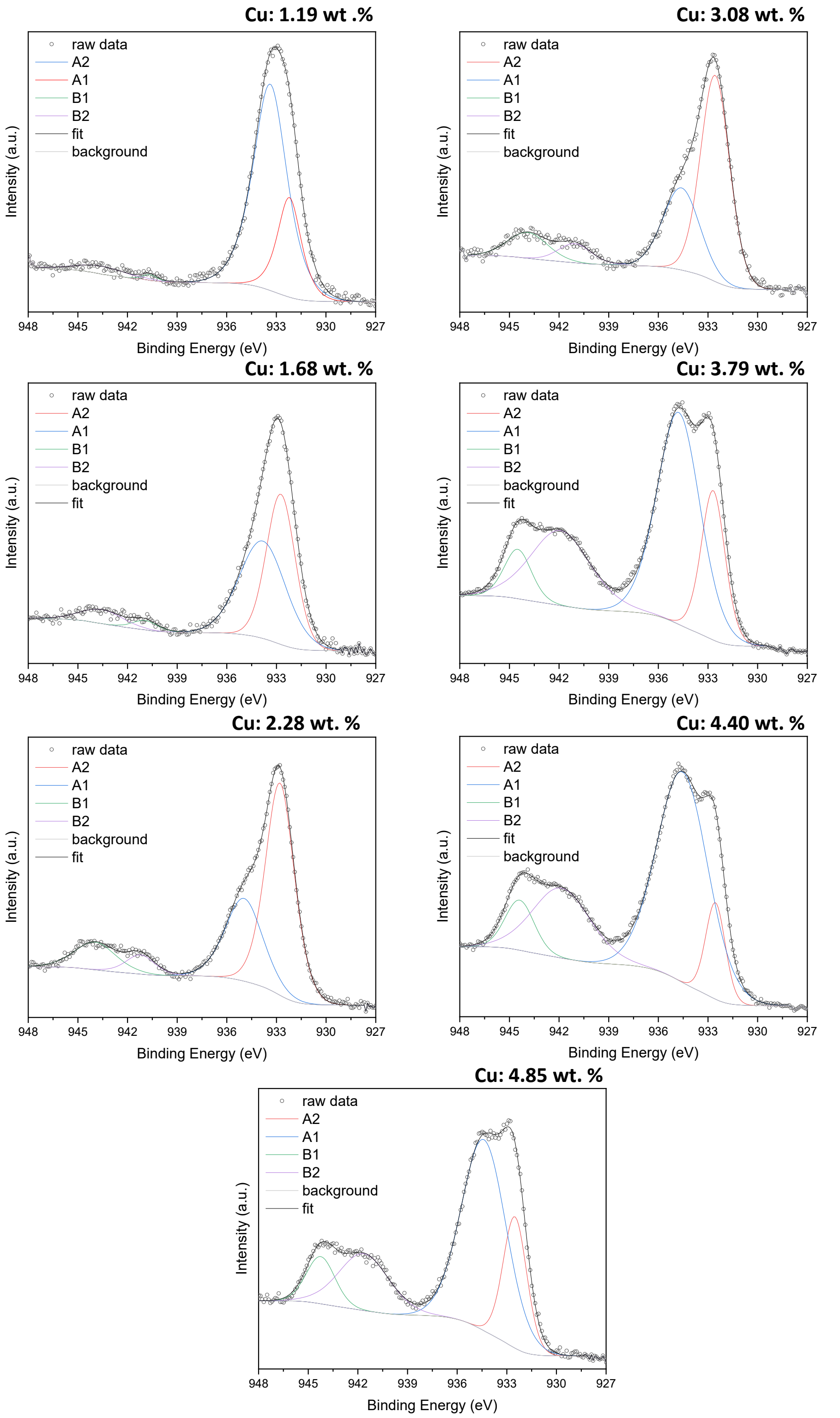

Figure S5. The deconvoluted copper $2p_{3/2}$ peaks and the corresponding satellite peaks of different ALD synthesized Cu_xO/TiO_2 samples.

Figure S6.

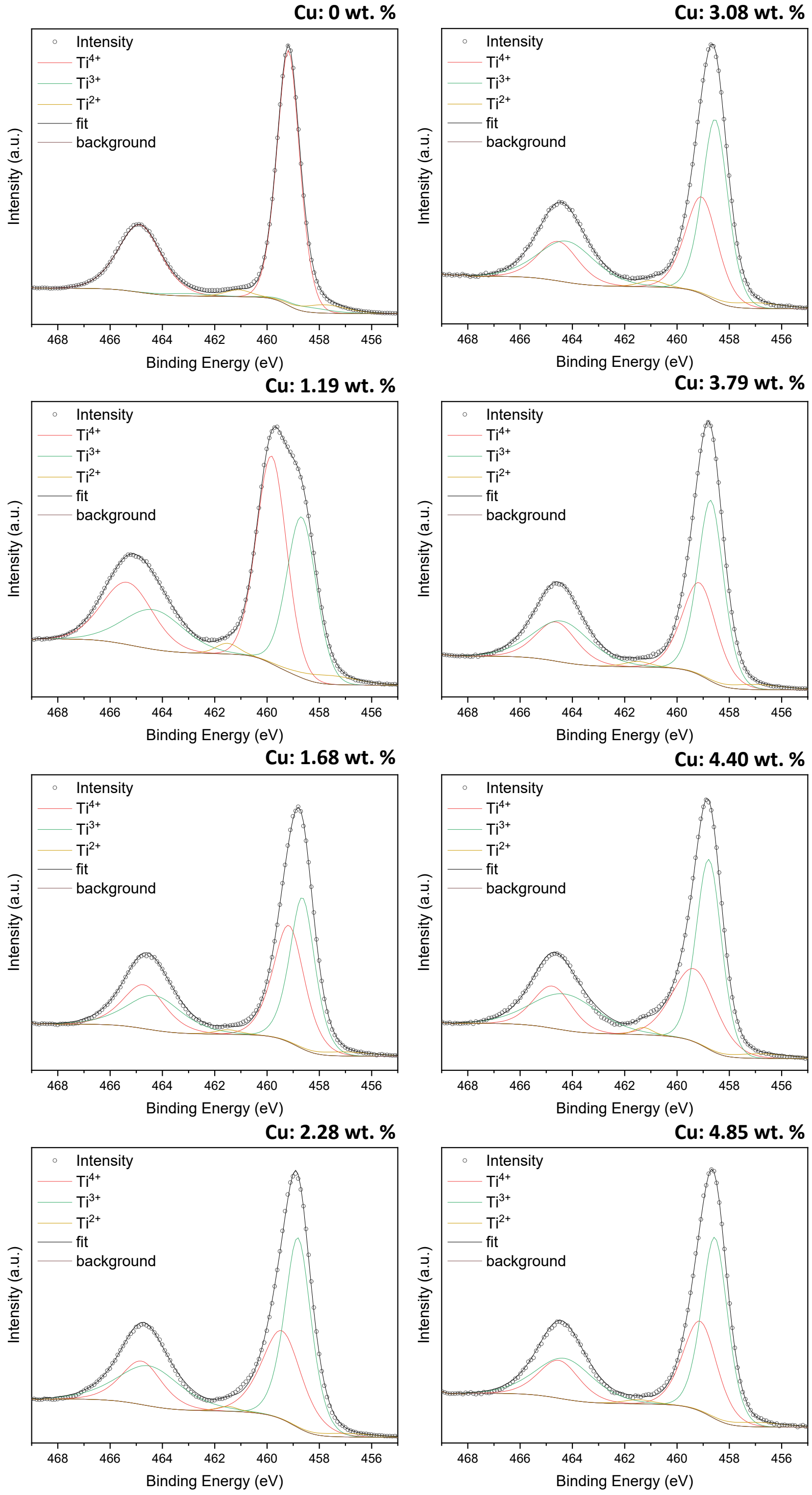

Figure S6. The deconvoluted titanium 2p spectra of pristine P25 TiO_2 and the different ALD synthesized Cu_xO/TiO_2 samples.

Figure S7.

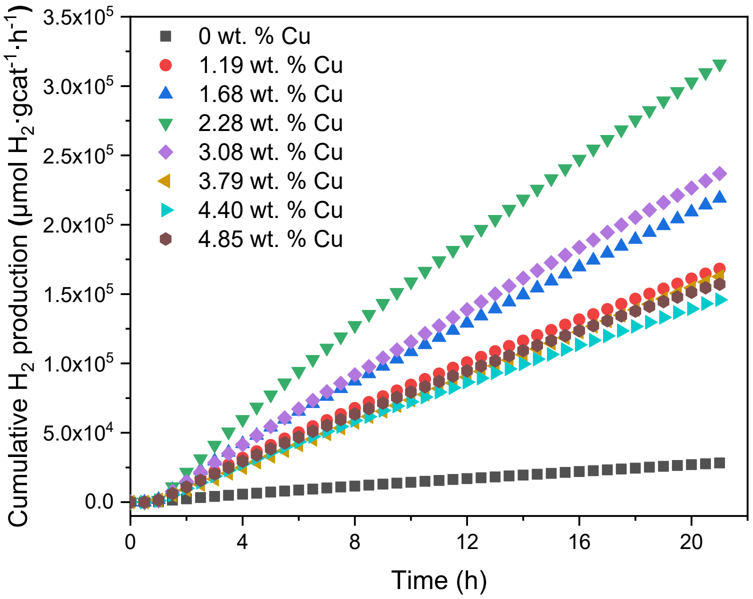

Figure S7. The cumulative hydrogen production after 20 hours of reaction as a function of copper loading in Cu_xO/TiO_2 photocatalysts.

Figure S8.

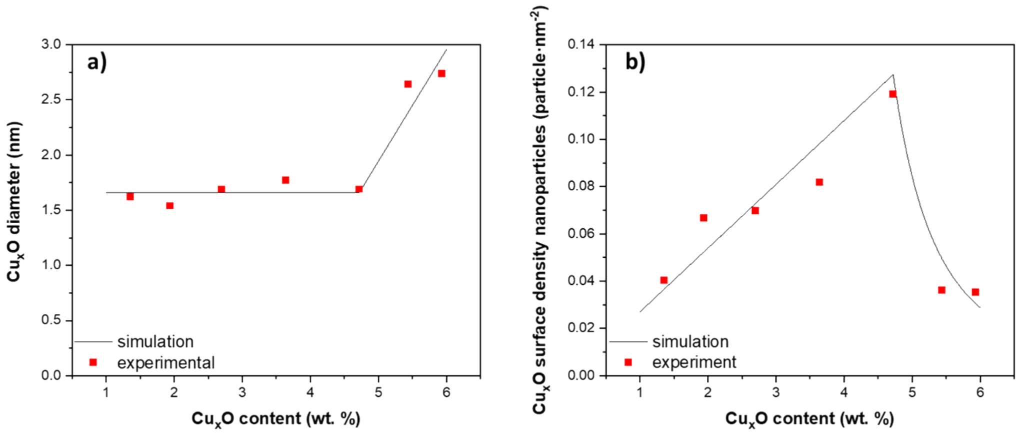

Figure S8. The average size (a) and the surface density (b) of Cu_xO particles. The squares show the experimental data, and the solid line indicates the function fitted to the experimental data.
